# Removal of Calcareous Concretions from Marine Archaeological Ceramics by Means of a Stimuli-Responsive Hydrogel

**DOI:** 10.3390/polym15132929

**Published:** 2023-07-02

**Authors:** Jianrui Zha, Qijun Huang, Xinyi Liu, Xiangna Han, Hong Guo

**Affiliations:** Institute of Cultural Heritage and History of Science and Technology, University of Science and Technology Beijing, Beijing 100083, China; b2159283@ustb.edu.cn (J.Z.); huangqj1117@126.com (Q.H.); m202221516@xs.ustb.edu.cn (X.L.)

**Keywords:** ceramics, gel cleaning, Ca^2+^ ion responsive, archeological information

## Abstract

The presence of calcareous concretions on the surface of marine archaeological ceramics is a frequently observed phenomenon. It is necessary to remove these materials when the deposits obscure the feature of ceramics. Unfortunately, calcareous concretions provide distinctive documentation of the burning history of ceramics. The interaction of acid solution or detachment of the deposit layers in physical ways leads to the loss of archeological information. To prevent the loss of archeological information and to achieve precise and gentle concretion removal, responsive hydrogel cleaning systems have been developed. The hydrogels synthesized are composed of networks of poly(vinyl acetate)/sodium alginate that exhibit desirable water retention properties, are responsive to Ca^2+^ ions, and do not leave any residues after undergoing cleaning treatment. Four distinct compositions were selected. The study of water retention properties involved quantifying the weight changes. The composition was obtained from Fourier transform infrared spectra. The microstructure was obtained from scanning electron microscopy. The mechanical properties were obtained from rheological measurements. To demonstrate both the efficiency and working mechanism of the selected hydrogels, a representative study of mocked samples is presented first. After selecting the most appropriate hydrogel composite, a cleaning process was implemented on the marine archaeological ceramics. This article demonstrates the advantages of stimuli-responsive hydrogels in controlling the release of acid solution release, thereby surpassing the limitations of traditional cleaning methods.

## 1. Introduction

Marine archaeological ceramics provide significant evidence for the technological development, social organization, and artistic evolution of ancient societies [1]. Nevertheless, they can present several alterations in the burial process, such as cracks, material loss, and concretions, which can impede archaeological investigations [2]. In the list of all alterations, the removal of concretions is a crucial procedure as these deposits obscure the feature of ceramics [3]. Key approaches, including light microscopy, X-ray diffraction, X-ray fluorescence, Raman spectroscopy, and Infrared spectroscopy, were employed to investigate the morphology and composition of concretions [4]. Calcium carbonate is typically composed due to its low solubility (CaCO_3_ kps = 4.8 × 10^−9^) and diversity of formation processes (such as from seabed sediments or shellfish) [5]. Calcareous concretions provide unique records of ancient biogeochemical processes in marine sediment and can be used to detect the burning history of ceramics [6]. So, for the removal of these deposits, conservators have developed and performed several methods based on the topic of how to effectively and controllably remove them [7]. As indicated by the reference, the predominant methods employed for the elimination of calcareous concretion were mechanical cleaning, physical cleaning, and chemical cleaning [8]. Mechanical cleaning treatments are accomplished using various tools, such as scalpels, scrapers, micro-drills, and micro-sandblasting, to gently remove concretions [9]. The efficacy of this method relies on the worker’s experience, the degree of concretion hardens, and the chosen analysis technique. Concretion removal by physical methods involves the use of pressure water/gas, ultrasonic radiation, laser ablation, or micro/nano bubble [10]. For chemical cleaning, the procedures generally consist of the direct use of chemical solutions or the application of poultices/gel cleaning systems [11]. Traditionally, diluted acids or chelating agents are the most common reagents used for the cleaning of ceramic materials. The dissolution mechanism of acid agents has been found in the concretion removal process [12]. Chelating agents effectively remove concretions using a surface complexation mechanism [13]. At the museum, conservators always utilize a combination of cleaning agents and various methods to effectively remove any possible concretions. It is important to bear in mind that the acid solution, which can effectively dissolve the concretions, may affect the original composition of the ceramics [14]. Various research has indicated that glazes containing iron oxides are prone to dissolution when subjected to acid treatment [15]. Moreover, during the immersion process, there is a possibility of recrystallization and deposition of ions from the acid and chelating solution after cleaning, which may pose a potential risk. To address this issue, it has been suggested that the hydrochloric acid solution cleaned sample should be rinsed with distilled water first, followed by immersion in a 10% solution of oxalic acid for a duration of 17 h. However, it should be noted that this method may result in the removal of archaeological information such as corrosion products [16]. In order to guarantee the safety of the ceramic and preserve more archaeological information, it is essential to improve the chemical cleaning process and further control it [17].

The gel cleaning method is considered a crucial technique for cleaning cultural heritage relics. It has been found to have several advantages over the chemical cleaning process, particularly in terms of providing greater control over the cleaning action on the surface of the sample [18]. This method has been used for the purpose of removing contaminants, conservation material, and effloresce products from different kinds of relics, including wall paintings, stone sculptures, paper, and metal artwork [19]. The fundamental constituents of the cleaning system comprised a hydrogel and a cleaning agent. Based on the removal object, the cleaning agent can be an organic solution, acid solution, alkaline solution, enzymes, surfactants, or chelating agent [20]. The hydrogel can function as a solution container and also exhibits a responsive behavior to external stimuli, especially pH, temperature, and magnetic fields. This feature provides conservators with a wide range of options [21]. Based on this, two classes of gel cleaning systems have been designed by conservators. (1) The inclusion of magnetic nanoparticles into polyacrylamide gels confers additional functionality to enable the loading of microemulsions or micellar structures, resulting in a versatile system. (2) The research of novel formulations of poly(vinyl alcohol)-borate gels that exhibit adequate strength for removal through the peeling method, as reported in reference [22].

Notwithstanding these properties, in the past, only a few works have discussed using partially hydrolyzed poly(vinyl acetate) (PVA) and borax, incorporated with chelators, to remove the gypsum degradation layers from carbonate matrices, and none of them have explored the application of this method for cleaning marine archaeological ceramics [23]. Indeed, they present some drawbacks in their potential application in calcareous concretion removal. For example, PVA-borax gels exhibit a high pH of approximately 9–10, rendering them unsuitable for accommodating acid solvents. This is due to the low pH levels of HCl solutions, which typically range from 1 to 3 and have concentrations from 5 to 25%. Additionally, while the hydrogel does not respond to the cleaning process, it should be improved to be more controllable for calcareous concretion removal [14,24]. The incorporation of a polymer into the PVA system as a substitute for borax and a responsive component has the potential to address the problem. Sodium alginate (SA) is a non-toxic polysaccharide extracted from brown seaweeds, extensively employed for the removal of various heavy metal ions from wastewater [25]. Most of the adsorbed ions were found on the cross-linked hydrogel structure, which had an impact on the release properties [26]. Inspired by this, an ion-responsive hydrogel composed of PVA, SA, and HCl was developed. Within these systems, the cross-linking agents of PVA and SA enable the formation of a homogeneous cross-linking network through a freeze-drying process. Additionally, the SA agent complexes with the dissolution ions (Ca^2+^) of calcareous concretion modulate the pore size of hydrogel, thereby controlling the reaction. The characterization of PVA/SA gels with different ratios was conducted using FTIR, SEM, and DSR techniques. Furthermore, an evaluation of their water-retentive properties was conducted. Moreover, the cleaning effectiveness was assessed through FTIR, SEM, and OM measurements on mock samples and marine archaeological ceramics.

## 2. Materials and Methods

### 2.1. Materials

Polyvinyl alcohol (PVA) (average Mw ≈ 1750, 97% hydrolyzed) and Hydrochloric acid (HCl) (assay 36%) were obtained from Sinopharm Chemical Reagent Co., Ltd (Shanghai, China). Sodium alginate (SA) (Viscosity ≈ 200 mpa.s) and calcium carbonate (CC) (assay 99%) were supplied by Shanghai Macklin Biochemical Co., Ltd. (Shanghai, China). Water was purified using a JRO-EDI-P19 gradient system.

The marine archaeological ceramic was sourced from (Shengbei yu, Zhangzhou, Fujian province, China) (Yuan dynasty). The entire surface of the sample is covered with a grey concretion. Samples are provided by the national center for archaeology.

### 2.2. Hydrogel Synthesis

Hydrogels with different concentrations of PVA and SA were prepared and subjected to testing. The gels were prepared following the protocol described in the literature. Initially, an appropriate amount of PVA and SA powder was dissolved in distilled water at 90 °C, resulting in an 8% (*w*/*v*) aqueous solution. Following a 12-h period, an aqueous solution of hydrochloric acid (pH = 1) was added, resulting in a final concentration of 25% (*V*/*V*). The aqueous mixture was introduced into a mold and subjected to a freeze/thaw cycle ranging from −20 °C to 20 °C. Table 1 presents the composition of the aforementioned hydrogel.

### 2.3. Calcium Carbonate Mockup

The objective of fabricating a calcium carbonate mockup in this study is to assess the cleaning mechanism of the hydrogel. A methodology was developed to prevent the formation of cracks on the surface of calcium carbonate. Initially, the calcite powder was introduced into a metal mold of 2 cm × 2 cm × 3 cm and compressed at a pressure of 30 Mpa for 1 min. Subsequently, square-shaped pieces were fired at a temperature of 500 °C to improve adhesion strength. To evaluate the efficacy of this method, the resulting pieces were immersed in a water bath for 30 min. The fired sample presented better mechanical properties compared to the unfired sample. Cleaning tests were performed after a 24-h cooling period. All mockups were stored in a dust-free environment.

### 2.4. Cleaning Treatments

Four mockup samples of calcium carbonate were cleaned using different hydrogel cleaning systems. A hydrogel piece measuring 1.75 cm × 3.5 cm (diameter × length) with a thickness of 5 mm was cut and then applied to the surface for 2 h. The hydrogel pieces were removed using forceps. After the completion of the treatments, it was imperative to assess the efficacy of each hydrogel cleaning system. The hydrogel with the best performance (PVA/SA_33) was applied to the surface of the marine archaeological ceramic surface for 2 h. After removing the gel using forceps, the cleaned area was characterized with a microscope.

### 2.5. Fourier Transform Infrared Spectrometer

The study employed a Thermo Fisher Nicolet iS 5 Fourier transform infrared spectrometer (FTIR) equipped with a DTGS detector to analyze the impact of acid solution and SA on the hydrogel. The results were obtained using an FTIR spectrophotometer within the 500–4000 cm^−1^ range, with a resolution of 16 scans. The hydrogel was freeze-dried, mixed with KBr powder through grinding, and then placed into the press mold for compression for 1 min.

Micro-reflectance Fourier transform infrared analyses were conducted on the samples utilized for hydrogel characterization and removal tests. The Thermo Fisher Nicolet in 10, equipped with a microscope for microanalysis, was employed for this purpose. A liquid nitrogen-cooled MCT detector was used to capture the signal within the 4000–650 cm^−1^ range, with a Fourier transform infrared spectral resolution of 4 cm^−1^ and 128 scans.

### 2.6. Microscopic Image

The microscopic images of the mock sample, marine archaeological ceramic, and hydrogel were captured using the VHX-6000 ultra-depth-of-field three-dimensional video microscope (Keyence, Osaka, Japan). The lens used was VH-ZST with a magnification of 50.

### 2.7. Scanning Electron Microscope

Scanning electron microscope (SEM) images were acquired using the Tescan Vega3 instrument for the hydrogel, as well as the surface and cross-sections of the cleaned sample. The hydrogel was subjected to freeze-drying and was then sectioned into smaller pieces. All samples were sputter-coated with gold and observed at an accelerating voltage of 20 kV.

### 2.8. Rheological Measurements

Rheological measurements were conducted on freshly prepared samples using a stress-controlled Anton Paar Physica MCR 302 rheometer, which was equipped with a 25 mm diameter cone (angle of 2.0°) and plate. Strain sweeps were measured over a range of 0.01–100% at a constant frequency of 10 Hz. Frequency sweeps were recorded in the range of 0.01–100 rad/s within the linear viscoelastic regions.

### 2.9. Water-Retention Properties

To express the water-retention properties of the hydrogel, the hydrogel films were accurately weighed and then transferred to a controlled laboratory environment for 21 h. A graphical representation in the form of a plot was constructed to illustrate the correlation between the hydrogel’s weight and the duration of time. Additionally, a fitted line was performed to better visualize this relationship.

## 3. Results and Discussion

### 3.1. Hydrogel Characterization

**Figure 1:**
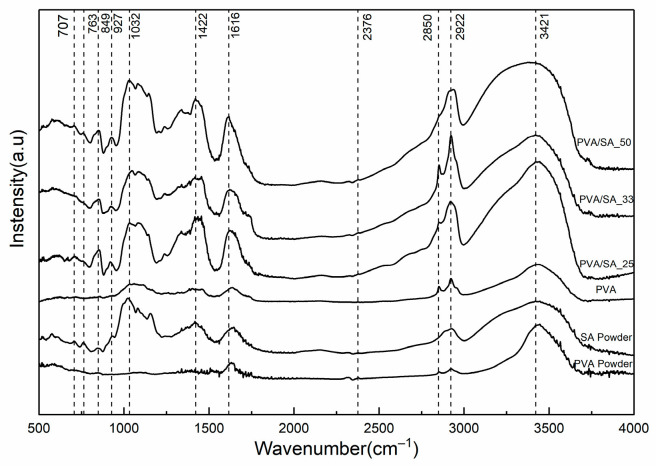
FTIR spectra of the powder (PVA, SA) and hydrogel (PVA, PVA/SA_25, PVA/SA_33, PVA/SA_50).

FTIR is commonly employed for identifying the main component of a hydrogel and detecting any additional compounds. The FTIR spectra of both PVA powder and PVA hydrogel display the characteristic peak of the PVA spectrum (Figure 1). It has been observed that PVA can effectively accommodate HCl solution without altering its functional group [27]. A thorough examination of the IR spectra of hydrogels with different SA content reveals slight differences between them. For instance, in the spectral region between 849 cm^−1^ and 1616 cm^−1^, a distinctive and complex pattern of SA transmission bands was observed, indicating the bending of Na-O, C-O and -COO bonds [28]. Additionally, there is another peak in this region that corresponds to the primary PVA peak (2850 cm^−1^, 2922 cm^−1^) [29], which indicates the physical bonding mechanism involved in hydrogel formation. Furthermore, it was observed that at a PVA/SA ratio of 2/1, the intensity of the -OH peaks at 3421 cm^−1^ significantly increased, suggesting a potential modification in water-retention properties.

**Figure 2:**
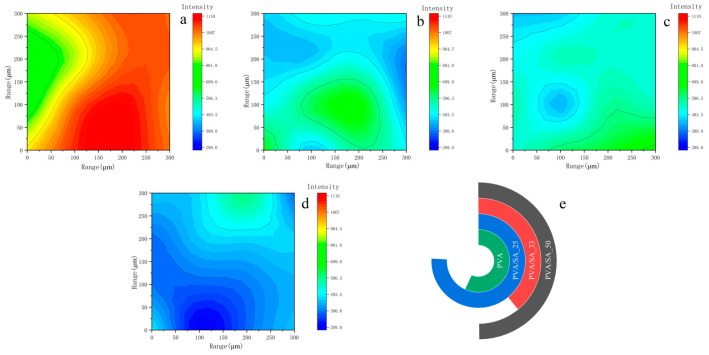
Hydrogel maps were obtained from the 2922 spectra that make up the FTIR map. (**a**) PVA; (**b**) PVA/SA_25; (**c**) PVA/SA_33; (**d**) PVA/SA_50; (**e**) measuring the similar color area in each FTIR map.

The 2D micro FTIR mapping was employed to confirm the precise location and distribution of the polymers on a micron scale [30]. The addition of sodium alginate, particularly at high concentrations, can disrupt the ordering of PVA chains, leading to a negative impact on the crystallinity, gelation, mechanical strength, and water uptake properties [31]. To examine any potential change in the distribution of the PVA chain in relation to the composition of the hydrogel, the primary PVA peak at 2922 cm^−1^ (C-H stretching) was captured via imaging [32]. The intensity of the band decreases significantly with increasing SA contents, as evidenced by the presence of green and blue pixels (no or low intensity). The statistic of the similar color area in each plot (Figure 2e) shows that the distribution of PVA chains is more uniform compared to the others at a 33% SA content.

**Figure 3:**
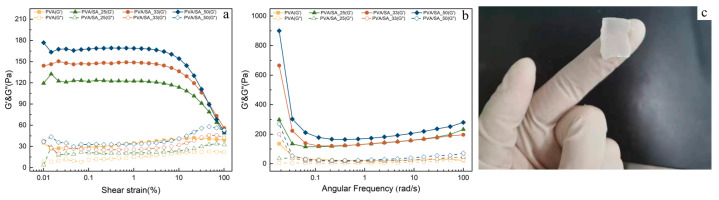
Amplitude sweep (**a**) and frequency sweep (**b**) curves of each hydrogel, (**c**) image of PVA/SA_33 hydrogel.

The rheological measurements enable us to establish the correlation between the mechanical properties and the addition of SA in the formulations [33]. Figure 3 illustrates the rheological behavior of each hydrogel. The relationship between the viscoelastic moduli and the applied shear strain is plotted in Figure 3a. The data indicate that an increase in SA concentration corresponds to higher moduli. These results can be attributed to the increased cross-linking density, leading to the formation of a highly structured polymer network with enhanced mechanical properties. Furthermore, within the investigated frequency range, it has been observed that the hydrogels exhibit a significant predominance of the storage modulus G′ above the loss modulus G″, demonstrating strong gel behavior [34]. The approach (without crossing) of G′ and G″ at the lowest frequency examined, 0.01 rad/s, suggests that the material is a soft gel with a long relaxation time [35]. The PVA/SA_33 hydrogels possess sufficient strength for cutting and, subsequently, cleaning.

**Figure 4:**
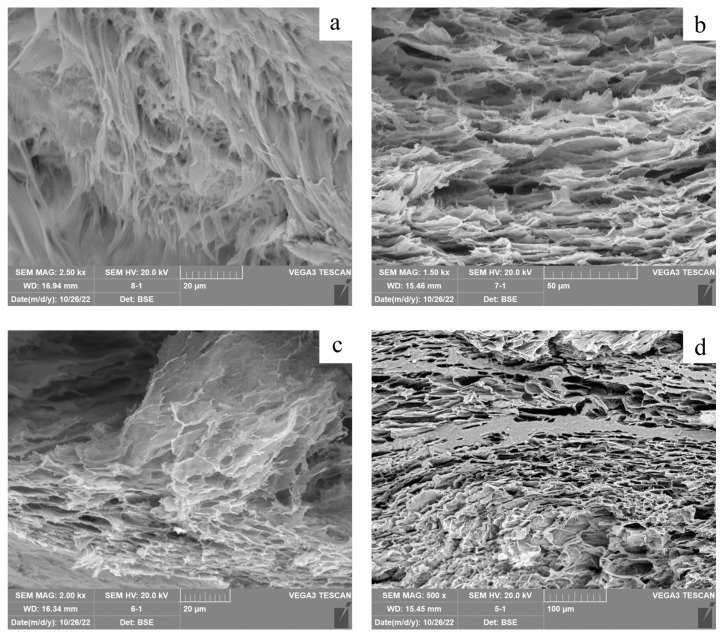
SEM image of hydrogel cross-section: (**a**) PVA, (**b**) PVA/SA_25, (**c**) PVA/SA_33, and (**d**) PVA/SA_50.

SEM images can provide detailed information about the structure and porosity of hydrogel [36]. Figure 4 confirms the uniform structure of the hydrogels. The image of the pure PVA hydrogels showed a rough surface morphology with a limited number of irregular pores. However, no discernible pores network was observed in the images. The incorporation of SA leads to the formation of a porous microstructure in the hydrogel. A noticeable change in the hydrogel pore network was observed when the SA content was increased to 33%. During the freezing stage, a cross-linked porous structure is formed after the phase separation of the mixed solution.

**Figure 5:**
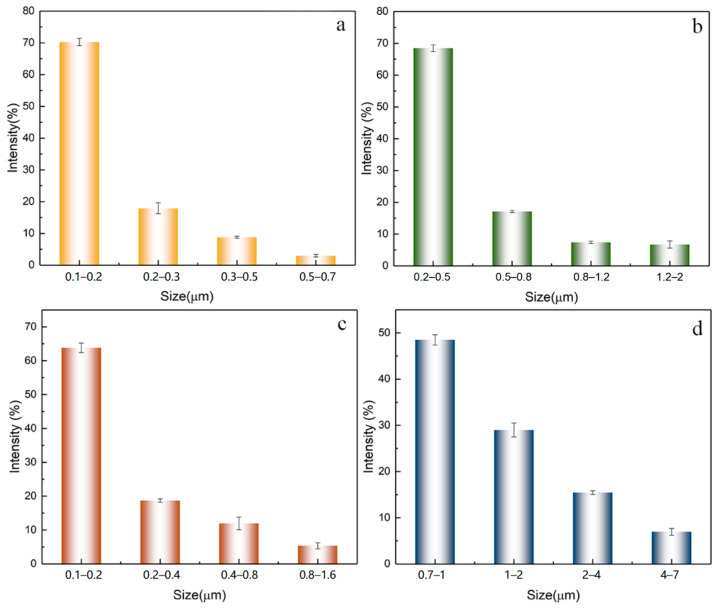
Pore size distribution of hydrogel (**a**) PVA, (**b**) PVA/SA_25, (**c**) PVA/SA_33, and (**d**) PVA/SA_50.

SEM images were analyzed for both hydrogels to extract a statistically significant pore size distribution using ImageJ software. The results are presented in Figure 5. It is evident that the PVA hydrogel exhibits a narrower distribution with characteristic dimensions ranging from 0.1 to 0.7 μm. Conversely, the PVA/SA hydrogel with 50% SA contents demonstrates a broader distribution, with a maximum pore diameter of approximately 7 μm. The main areas of pore sizes distribution extracted within each hydrogel are 0.1–0.2 0.2–0.5, 0.1–0.2, and 0.7–1. The variation in pore size distribution can be attributed to the volume fraction of the polymeric precursors. A sharp increase in pore size is observed from the PVA/SA_33 to the PVA/SA_50 case, likely due to a higher concentration of SA dissolved in the HCl solution.

**Figure 6:**
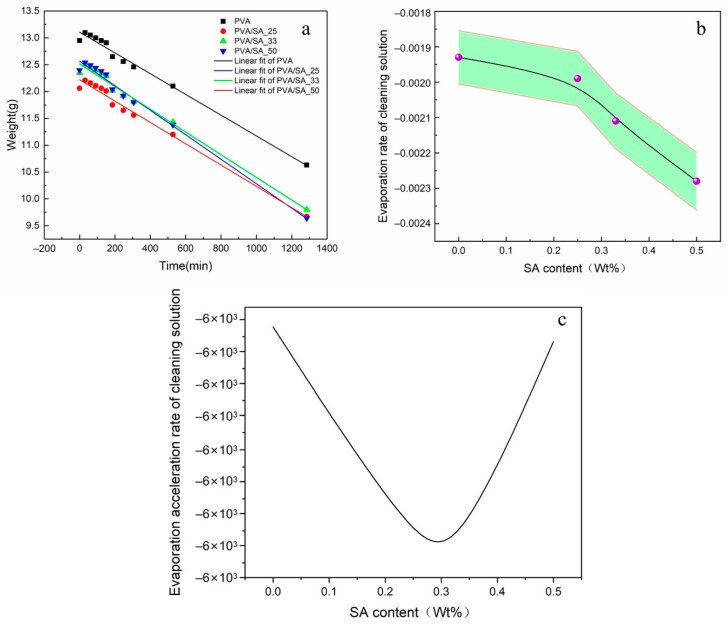
(**a**) Hydrogel weight change as a function of time. (**b**) Evaporation rate of solution as a function of the SA content (%). (**c**) The acceleration of evaporation rate as a function of the SA content (%).

Dehydration curves of PVA, PVA/SA_25, PVA/SA_33, and PVA/SA_ 50 hydrogels are displayed in Figure 6. Both hydrogels exhibited a uniform water release pattern. After testing for 1260 min, it is evident that the increase in evaporation rate aligns with the changes in the pore size structure (Figure 6b). In order to validate the effect of SA content on solution release, particularly if it alters during the cleaning process due to Ca^2+^ response, a functional plot illustrating the relationship between the acceleration of evaporation rate and SA content was generated. The minimum point on the curve is positioned at 0.3, which corresponds to the composition of PVA/SA_33. This finding confirms that the PVA/SA_33 case offers greater control during the cleaning process.

### 3.2. Gel Cleaning Tests

**Figure 7:**
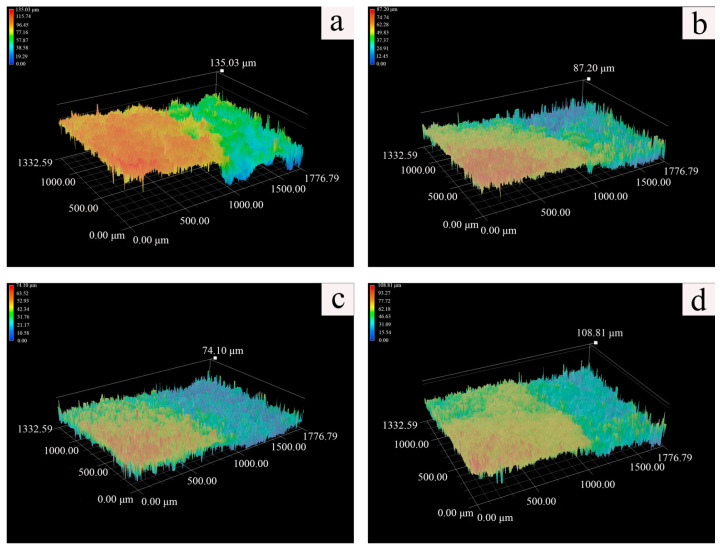
**A** 3D photomicrograph showing the boundary between a treated and an untreated area of the mocked sample treated with four hydrogel composites: (**a**) PVA, (**b**) PVA/SA_25, (**c**) PVA/SA_33, and (**d**) PVA/SA_50.

All the hydrogel formulations described in Table 1 were tested to evaluate their cleaning performance when applied to the mocked sample. Results obtained from photographs/height maps captured using 3D microscopy were found to align with the hydrogel water-retention test result. The mock sample cleaned with PVA/SA_33 exhibited a relatively smooth surface topography (Figure 7c), while the mock sample treated with PVA hydrogel displayed rough protrusion structures in the cleaned area (green to yellow region), resembling the cases of PVA/SA_25, and PVA/SA_50 (Figure 7b,d). The addition of SA facilitates the formation of cross-link networks and improves the controllability of the cleaning process in the hydrogel.

**Figure 8:**
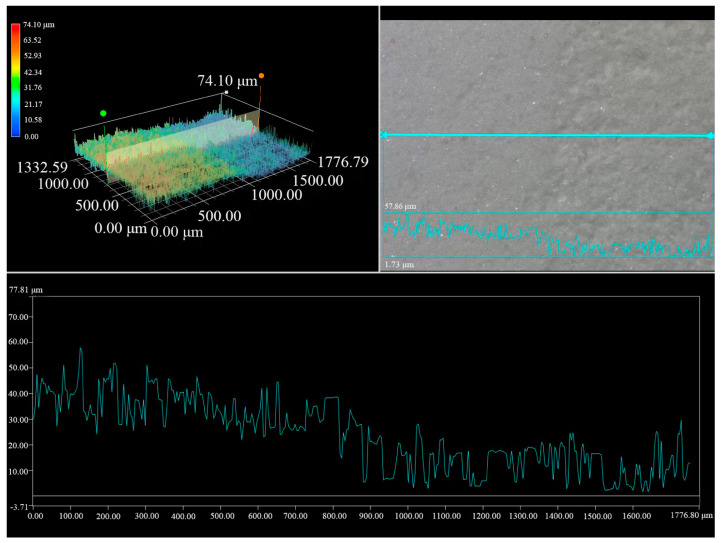
Liner scanning results of the mocked sample treated with hydrogel PVA/SA_33.

The thickness of the removed layer in the PVA/SA_33 hydrogel-treated sample was approximately 50 μm, showing reduced variation (Figure 8). Additionally, it is worth noting that the thickness of concretion discovered in marine archaeological relics is generally thicker than this [37]. Furthermore, the amount of thickness removed decreases with the duration of hydrogel application, indicating the versatility of the hydrogel. These results suggest that the hydrogel can effectively and gently remove calcareous concretion.

**Figure 9:**
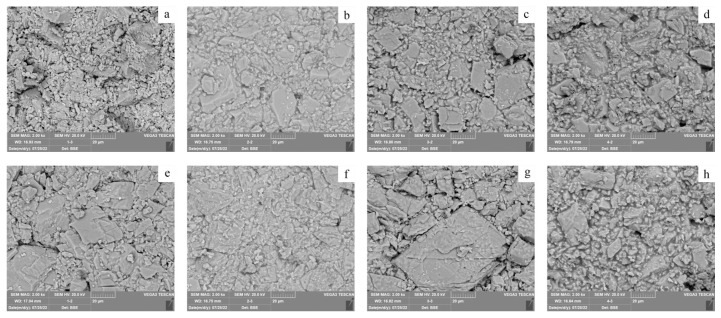
Sem image showing the untreated and treated area of the mock samples treated with four hydrogel composites: (**a**) untreated area of PVA, (**b**) untreated area of PVA/SA_25, (**c**) untreated area of PVA/SA_33, (**d**) untreated area of PVA/SA_50, (**e**) treated area of PVA, (**f**) treated area of PVA/SA_25, (**g**) treated area of PVA/SA_33, and (**h**) treated area of PVA/SA_50.

We imaged the untreated and treated areas using SEM, as shown in Figure 9. SEM images revealed that the use of hydrogel for cleaning purposes does not leave any residues on both mock samples. In addition, the absence of chloride (Cl) residues in the cleaned samples is supported by the data presented in Figure S1 and Table S1, as discussed in the introduction. Furthermore, microscopic images allow us to observe that the cleaning process is effective, as seen in the clearer appearance of the PVA/SA_33 hydrogel-treated sample, which exhibits an external surface phenomenon. Both etching pits grow an island, and slight development of grain boundary widening was detected, which may be attributed to the combined action of the proton (HCl) and chelating (SA) process [38].

**Figure 10:**
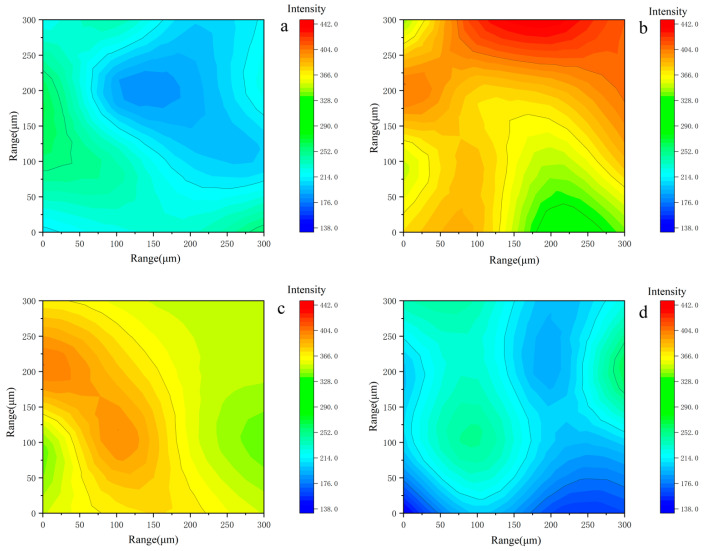
Mocked sample maps obtained from the 711 spectra that make up the FTIR map. (**a**) After PVA clean, (**b**) after PVA/SA_25 clean, (**c**) after PVA/SA_33 clean, and (**d**) after PVA/SA_50 clean.

The results of the IR mapping of the treated area are illustrated in Figure 10. The intensity of the functional group indicates distinct cleaning processes of the hydrogel. For instance, the PVA sample exhibits a low intensity of the C-O bond compared to the PVA/SA_33 sample. Consequently, the former displays a higher concentration of calcium (Ca) atoms on the surface of the grains. These characteristics align with the introduction of SA to facilitate the complexation with Ca ions and regulate the cleaning process.

**Figure 11:**
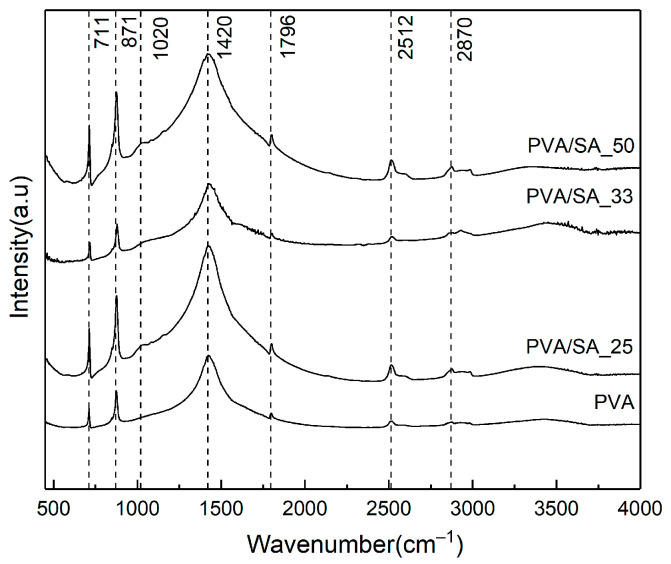
FTIR spectra of used hydrogel (PVA, PVA/SA_25, PVA/SA_33, PVA/SA_50).

All hydrogels were characterized through FTIR spectroscopy (Figure 11) after cleaning. Analysis of the FTIR spectra revealed a decrease in the intensity of -OH stretching vibration after the cleaning process, indicating the significant role of the solution in the cleaning process. Furthermore, various peaks were observed for PVA, including 2512 cm^−1^ (HCO_3_^−^), 1796 cm^−1^ (C=O), 1420 cm^−1^ (COO-), 871 cm^−1^ (Ca-O), and 711 cm^−1^ (C-O) [39]. Similar results were obtained for the PVA/SA_25, PVA/SA_33, and PVA/SA_50 samples. Notably, the presence of a peak at 1020 cm^−1^ (SA-Ca) [40] indicates a chelation process, confirming the combined effect of the solution and hydrogel. Furthermore, the intensity of the characteristic peak of CaCO_3_ sharply decreased in the PVA/SA_33 sample, suggesting that the removed concretion layer was relatively thinner than the other samples.

**Figure 12:**
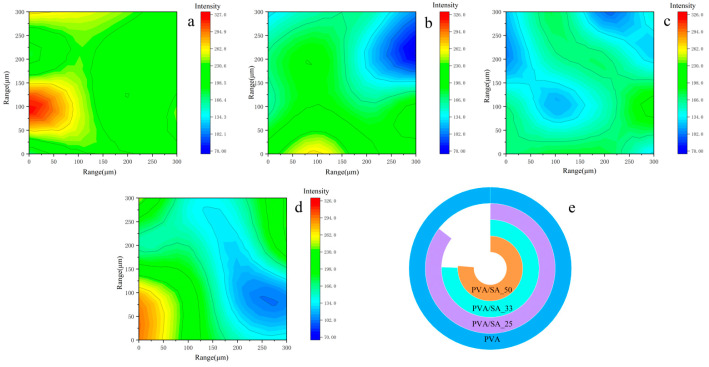
Used hydrogel maps obtained from the 711 spectra that make up the FTIR map. (**a**) PVA, (**b**) PVA/SA_25, (**c**) PVA/SA_33, (**d**) PVA/SA_50, and (**e**) measuring the green/yellow/red area in each FTIR map.

Additional insights could be obtained by conducting a corresponding 2D-IR analysis of the hydrogels after cleaning at a wavenumber of 711 cm^−1^ to remove CaCO_3_ (Figure 12). The maps show a low presence of CaCO_3_, represented by blue pixels. Red or yellow pixels indicate a weak intensity of C-O stretching absorptions, whereas green pixels indicate a medium intensity range. Figure 12 demonstrates that adding SA to the hydrogel significantly decreased the thickness of the removed layer. As expected, the statistical results reveal that the PVA/SA_33 case produced the largest area of blue.

**Figure 13:**
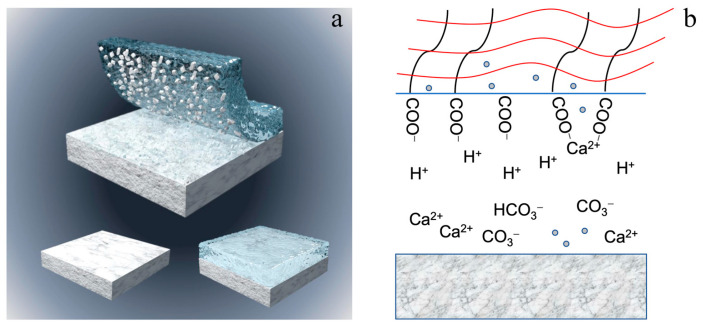
(**a**) General view of the hydrogel cleaning process. (**b**) Cleaning mechanism of the hydrogel.

The cleaning processes are depicted in Figure 13. Figure 13a shows representative images of the general view of the hydrogel cleaning process. The interaction of the hydrogel with the mocked sample results in a combined chemical dissolution and physical adsorption process. Figure 13b demonstrates the representative cleaning mechanism of the hydrogel. In summary, the presence of SA-Ca inside PVA/SA_25, PVA/SA_33, and PVA/SA_50 gels after cleaning can be attributed to the stimulus-response of dissolution ions (Ca^2+^), resulting in a modification of the hydrogel’s pore size. 

**Figure 14:**
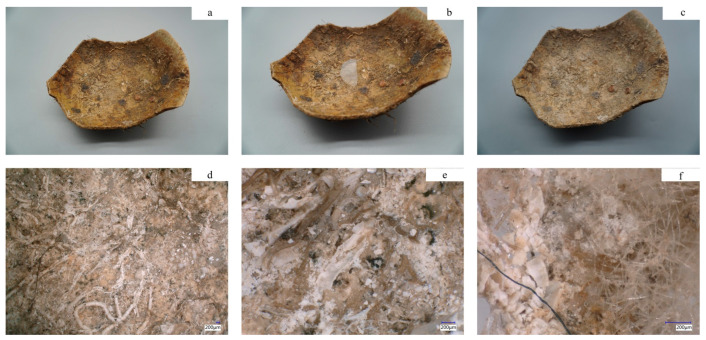
(**a**) General view of the archaeological sample for the cleaning test. (**b**) The hydrogel is applied to the surface. (**c**) General view of the archaeological sample after gel cleaning treatment. (**d**) Microscopy image of the cleaning area (20×). (**e**) The same area under higher magnification (100×). (**f**) Microscopy image of the cleaned hydrogel surface.

The marine archaeological ceramic dating from 1271 to 1368 AD is a green glaze from the Longquan Kiln that was evacuated in Fujian province. The sample surface is covered in concretions of marine life wrecks. In this study, the concretion at the center of the sample surface was removed using Hydrogel PVA/SA_33, as depicted in Figure 14. As a result of the treatment, the previously obscured section of the cleaning area became visible, revealing surface-level coral crushing (Figure 14d). It is worth noting that no discernible damage was observed in the underlying layer (Figure 14e). Furthermore, the hydrogel successfully removed the other composition concretion in the selected test areas (Figure 14f).

## 4. Conclusions

The PVA/SA hydrogel was found to be effective in removing calcareous concretion from marine archaeological ceramics. The hydrogel shows the ability to respond to the cleaning process and enables the consistent release of acid solution during the dissolution process. For archaeological samples, the hydrogel can be controlled effectively to remove concretions without affecting the inner layer. Among the tested cleaning systems, PVA/SA_33 exhibited the highest level of control in removing calcareous concretion. This difference can be attributed to the distinct structures and water retention properties of the hydrogels. Further investigations of the hydrogel aim to design and assess the effectiveness of magnetic response hydrogel in removing iron deposits from marine archaeological surfaces.

## Figures and Tables

**Table 1:** Composition of the PVA/SA hydrogel.

Name	Polymer	PVA (g)	SA (g)	H_2_O (mL)	HCl (mL)
PVA	PVA	8	0	92	25
PVA/SA_25	PVA/SA = 3/1	6	2	92	25
PVA/SA_33	PVA/SA = 2/1	5.33	2.66	92	25
PVA/SA_50	PVA/SA = 1/1	4	4	92	25

## Data Availability

Not applicable.

## Supplementary Materials

The following supporting information can be downloaded at: https://www.mdpi.com/article/10.3390/polym15132929/s1, Figure S1: SEM results of treated and untreated area of mocked samples. Table S1: EDS results of treated and untreated areas of mocked samples.

## References

[1] Hamilton D.L. (1999). Conservation of cultural materials from underwater sites. Arch. Mus. Inform..

[2] He Y., Li W., Li J., Xu C., Lu X. (2021). Corrosion of Longquan celadons in the marine environment: Study on the celadons from the Dalian Island shipwreck of the Yuan Dynasty. Herit. Sci..

[3] Dong J., Li Q., Liu S. (2020). Scientific analysis of some glazed pottery unearthed from Warring States Chu tombs in Jiangling, Hubei Province: Indication for the origin of the low-fired glazed pottery in China. X-ray Spectrom..

[4] Hao X.-L., Zhu T.-Q., Xu J.-J., Wang Y.-R., Zhang X.-W. (2018). Microscopic study on the concretion of ceramics in the “Nanhai I” shipwreck of China, Southern Song Dynasty (1127–1279 A.D.). Microsc. Res. Tech..

[5] Morse J.W., Arvidson R.S., Lüttge A. (2007). Calcium carbonate formation and dissolution. Chem. Rev..

[6] Gaines R.R., Vorhies J.S. (2016). Growth mechanisms and geochemistry of carbonate concretions from the Cambrian Wheeler Formation (Utah, USA). Sedimentology.

[7] Zhang Z., Ma Q., Li N., Tian X. Research on the removal of calcareous and iron concretions from marine finds. Proceedings of the 2014 Asia-Pacific Regional Conference on Underwater Cultural Heritage.

[8] Wang Y., Zhu T., Yang G., Tan X., Ye D., Chen H. (2018). The method to soften the concretions of ceramics in the “Nanhai I” Shipwreck of China Southern Song Dynasty (1127–1279AD). Herit. Sci..

[9] Turner-Walker G. The nature of cleaning: Physical and chemical aspects of removing dirt, stains and corrosion. Proceedings of the international symposium on cultural heritage conservation.

[10] Huet N., Tanguy E., Vincotte A., Zafiropulos V. Using lasers for cleaning ceramic and plaster patrimonial objects. Proceedings of the SPIE—The International Society for Optical Engineering.

[11] Coladonato M., Di Odoardo B., Prunas E. (2013). Removal of calcareous concretions from natural and manufactured stone archaeological artefacts through the use of CO_2_ water solutions. Procedia Chem..

[12] Baglioni M., Giorgi R., Berti D., Baglioni P. (2012). Smart cleaning of cultural heritage: A new challenge for soft nanoscience. Nanoscale.

[13] Sáenz-Martínez Á., Pérez-Estébanez M., de Buergo M.A., Andrés M.S. (2023). Chelating agents for the removal of calcareous deposits from archaeological ceramic materials. Compositional evaluation after immersion and physical gel application methods. Eur. Phys. J. Plus.

[14] Johnson J.S., Erickson H.M., Iceland H. (1995). Identification of chemical and physical change during acid cleaning of ceramics. MRS Proc..

[15] Sáenz-Martínez Á., Andrés M.S., de Buergo M.A., Blasco I., Fort R. (2019). Removing calcium carbonate deposits from archaeological ceramics. traditional methods under review. Mediterr. Archaeol. Archaeom..

[16] Hamilton D.L. (1996). Basic Methods of Conserving Underwater Archaeological Material Culture.

[17] Fritz-Endres T., Fehrenbacher J. (2021). Preferential loss of high trace element bearing inner calcite in foraminifera during physical and chemical cleaning. Geochem. Geophys. Geosyst..

[18] Baglioni P., Berti D., Bonini M., Carretti E., Perez M.D.C.C., Chelazzi D., Dei L., Fratini E., Giorgi R., Natali I. (2012). Gels for the conservation of cultural heritage. Langmuir.

[19] Baglioni M., Poggi G., Chelazzi D., Baglioni P. (2021). Advanced materials in cultural heritage conservation. Molecules.

[20] Baglioni P., Baglioni M., Bonelli N., Chelazzi D., Giorgi R. (2019). Smart Soft Nanomaterials for Cleaning. Nanotechnologies and Nanomaterials for Diagnostic, Conservation and Restoration of Cultural Heritage.

[21] Yang Z., Chen L., McClements D.J., Qiu C., Li C., Zhang Z., Miao M., Tian Y., Zhu K., Jin Z. (2022). Stimulus-responsive hydrogels in food science: A review. Food Hydrocoll..

[22] Carretti E., Bonini M., Dei L., Berrie B.H., Angelova L.V., Baglioni P., Weiss R.G. (2010). New frontiers in materials science for art conservation: Responsive gels and beyond. Acc. Chem. Res..

[23] Berlangieri C., Andrina E., Matarrese C., Carretti E., Traversi R., Severi M., Chelazzi D., Dei L., Baglioni P. (2017). Chelators confined into 80pvac-borax highly viscous dispersions for the removal of gypsum degradation layers. Pure Appl. Chem..

[24] Mazzuca C., Severini L., Domenici F., Toumia Y., Mazzotta F., Micheli L., Titubante M., Di Napoli B., Paradossi G., Palleschi A. (2020). Polyvinyl alcohol based hydrogels as new tunable materials for application in the cultural heritage field. Colloids Surf. B Biointerfaces.

[25] Alqadami A.A., Khan M.A., Siddiqui M.R., Alothman Z.A., Sumbul S. (2020). A facile approach to develop industrial waste encapsulated cryogenic alginate beads to sequester toxic bivalent heavy metals. J. King Saud Univ. Sci..

[26] Roger S., Talbot D., Bee A. (2006). Preparation and effect of Ca^2+^ on water solubility, particle release and swelling properties of magnetic alginate films. J. Magn. Magn. Mater..

[27] Jegal J., Oh N.-W., Park D.-S., Lee K.-H. (2001). Characteristics of the nanofiltration composite membranes based on PVA and sodium alginate. J. Appl. Polym. Sci..

[28] Derkach S.R., Voron’ko N.G., Kuchina Y.A. (2022). Intermolecular Interactions in the Formation of Polysaccharide-Gelatin Complexes: A Spectroscopic Study. Polymers.

[29] Pal K., Banthia A.K., Majumdar D.K. (2007). Preparation and characterization of polyvinyl alcohol-gelatin hydrogel membranes for biomedical applications. AAPS Pharmscitech.

[30] Baglioni M., Alterini M., Chelazzi D., Giorgi R., Baglioni P. (2019). Removing polymeric coatings with nanostructured fluids: Influence of substrate, nature of the film, and application methodology. Front. Mater..

[31] Adelnia H., Ensandoost R., Moonshi S.S., Gavgani J.N., Vasafi E.I., Ta H.T. (2022). Freeze/thawed polyvinyl alcohol hydrogels: Present, past and future. Eur. Polym. J..

[32] El-Attar A.A., El-Wakil H.B., Hassanin A.H., Bakr B.A., Almutairi T.M., Hagar M., Elwakil B.H., Olama Z.A. (2022). Silver/Snail mucous PVA nanofibers: Electrospun synthesis and antibacterial and wound healing activities. Membranes.

[33] Riedo C., Caldera F., Poli T., Chiantore O. (2015). Poly (vinylalcohol)-borate hydrogels with improved features for the cleaning of cultural heritage surfaces. Herit. Sci..

[34] Bertasa M., Dodero A., Alloisio M., Vicini S., Riedo C., Sansonetti A., Scalarone D., Castellano M. (2020). Agar gel strength: A correlation study between chemical composition and rheological properties. Eur. Polym. J..

[35] Duncan T.T., Berrie B.H., Weiss R.G. (2017). Soft, Peelable Organogels from Partially Hydrolyzed Poly(vinyl acetate) and Benzene-1,4-diboronic Acid: Applications to Clean Works of Art. ACS Appl. Mater. Interfaces.

[36] Domingues J.A.L., Bonelli N., Giorgi R., Fratini E., Gorel F., Gorel F. (2013). Innovative hydrogels based on semi-interpenetrating p(HEMA)/PVP networks for the cleaning of water-sensitive cultural heritage artifacts. Langmuir.

[37] Domoney K. (2009). The Gresham Ship, Thames Estuary: Conservation of an Elizabethan Shipwreck Assemblage.

[38] Ruiz-Agudo E., Kudłacz K., Putnis C.V., Putnis A., Rodriguez-Navarro C. (2013). Dissolution and carbonation of portlandite [Ca (OH)_2_] single crystals. Environ. Sci. Technol..

[39] Qin Z., Ren X., Shan L., Guo H., Geng C., Zhang G., Ji S., Liang Y. (2016). Nacrelike-structured multilayered polyelectrolyte/calcium carbonate nanocomposite membrane via Ca-incorporated layer-by-layer-assembly and CO_2_-induced biomineralization. J. Membr. Sci..

[40] Yue Y., Han J., Han G., French A.D., Qi Y., Wu Q. (2016). Cellulose nanofibers reinforced sodium alginate-polyvinyl alcohol hydrogels: Core-shell structure formation and property characterization. Carbohydr. Polym..

