# Supplementary material for: Removal of Calcareous Concretions from Marine Archaeological Ceramics by Means of a Stimuli-Responsive Hydrogel
Source: Polymers (Basel). 2023 Jul 2;15(13):2929. doi: 10.3390/polym15132929 (PMC10346492; doi:10.3390/polym15132929)
Supplement: Supplementary file 1 [file polymers-15-02929-s001.zip › polymers-2434004-supplementary.pdf]

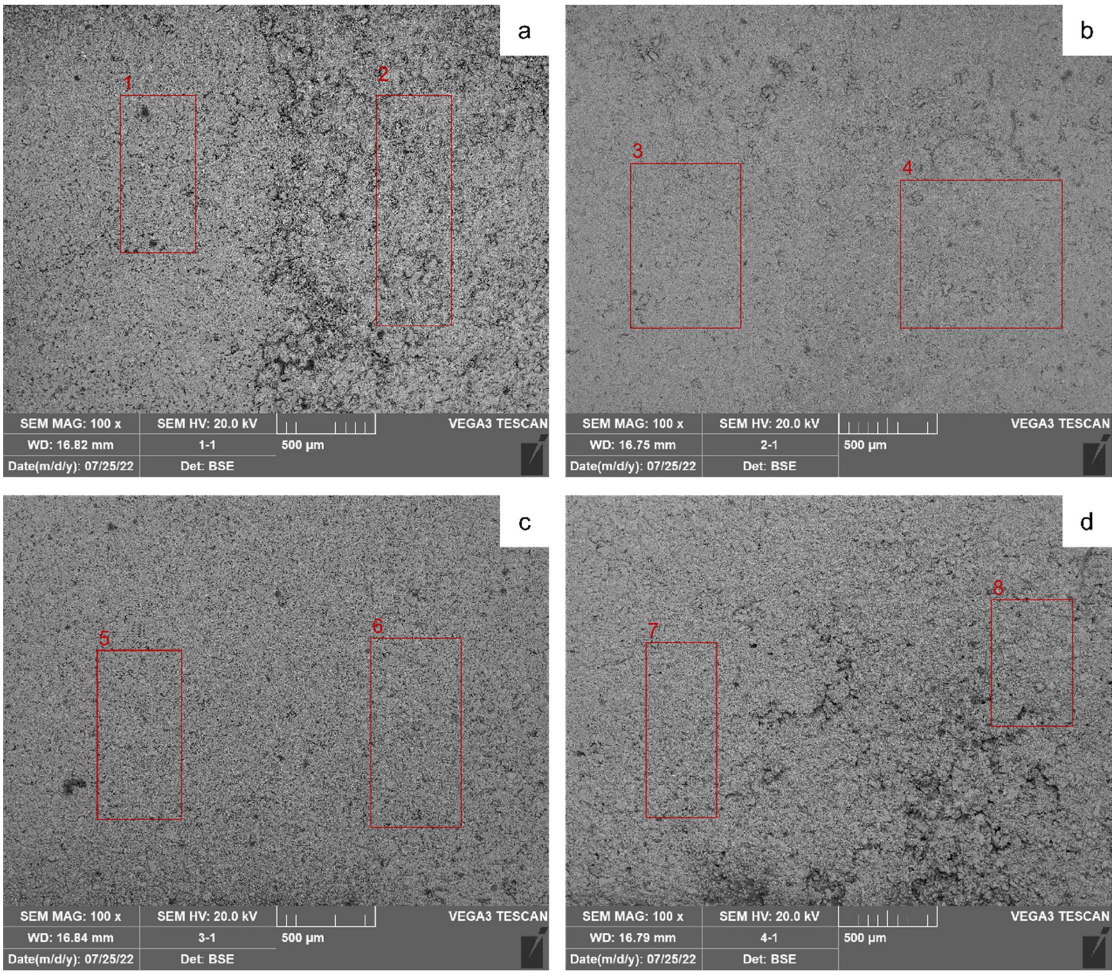

Figure S1: SEM results of treated and untreated area of mocked samples

Table S1: EDS results of treated and untreated areas of mocked samples（%）

Code	O	Ca
1	51.2	48.8
2	52.3	47.7
3	50.2	49.8
4	52.3	47.7
5	50.5	49.5
6	51.4	48.6
7	52.1	49.8
8	51.5	47.7
